# Low grip strength and gait speed as markers of dependence regarding basic activities of daily living: the FIBRA study

**DOI:** 10.31744/einstein_journal/2024AO0637

**Published:** 2024-05-09

**Authors:** Juliana Carvalho Segato Marincolo, Daniela de Assumpção, Mariana Reis Santimaria, Ivan Aprahamian, Mônica Sanches Yassuda, Anita Liberalesso Neri, Ligiana Pires Corona, Flávia Silva Arbex Borim

**Affiliations:** 1 Postgraduate Program in Gerontology Universidade Estadual de Campinas Campinas SP Brazil Postgraduate Program in Gerontology, Universidade Estadual de Campinas, Campinas, SP, Brazil.; 2 Faculdade de Fisioterapia Pontifícia Universidade Católica de Campinas Campinas SP Brazil Faculdade de Fisioterapia, Pontifícia Universidade Católica de Campinas, Campinas, SP, Brazil.; 3 Department of Internal Medicine Faculdade de Medicina de Jundiai Jundiai SP Brazil Group of Investigation on Multimorbidity and Mental Health in Aging, Department of Internal Medicine, Faculdade de Medicina de Jundiai, Jundiai, SP, Brazil.; 4 Department of Psychiatry University of Groningen Groningen Netherlands Department of Psychiatry, University of Groningen, Groningen, Netherlands.; 5 Postgraduate Program in Gerontology Escola de Artes, Ciências e Humanidades Universidade de São Paulo São Paulo SP Brazil Postgraduate Program in Gerontology, Escola de Artes, Ciências e Humanidades, Universidade de São Paulo, São Paulo, SP, Brazil.

**Keywords:** Activities of daily living, Aged, Walking speed, Gait, Muscle strength, Risk factor

## Abstract

**Objective:**

To determine whether low muscle strength and low gait speed increase the risk of disability related to basic activities of daily living in community-dwelling older adults.

**Methods:**

A longitudinal study (9 years of follow-up) was conducted with 390 older adults who were independent in basic activities of daily living at baseline and answered the Katz Index at follow-up. Associations were determined using Pearson’s χ^2^ test with a 5% significance level and logistic regression analysis.

**Results:**

Increases in prevalence between baseline and follow-up were observed for low muscle strength (17.5%-38.2%), slow gait speed (26.0%-81.1%), and functional dependence (10.8%-26.6%). At follow-up, 11.7% of the participants had concomitant functional dependence, slow gait speed, and low muscle strength. Slow gait speed remained a predictor of dependence in basic activities of daily living (odds ratio=1.90; 95% confidence interval=1.06-3.41).

**Conclusion:**

Slow gait speed is a predictor of functional dependence, constituting an important variable for screening functional decline.

## In Brief

Marincolo et al. showed that older adults without limitations in basic activities of daily living at baseline presented with an 11.7% concomitant presence of functional dependence, slow gait speed, and low muscle strength at follow-up. Slow gait speed remains a predictor of dependence in basic activities of daily living.

## Highlights

Atotal of 11.7% had concomitant limitations in activities ofdaily living, slow gait speed, and low muscle strength.

Grip strength was not associated with limitations in activities of daily living.

Slow gait speed increases the likelihood of limitations in activities of daily living.

Gait speed is an essential variable for screening functional decline in older people.

## Graphical abstract

**Figure:**
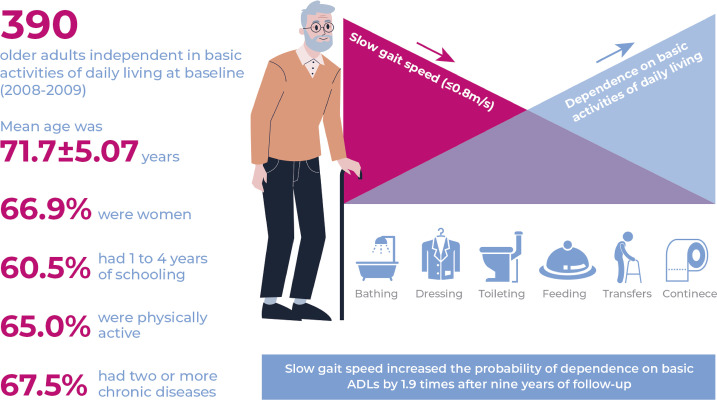

## INTRODUCTION

Biological markers such as grip strength and gait speed are considered important indicators of muscle strength and physical performance and are associated with the level of functional capacity in older people.^(1-3)^ The literature describes declines in these markers as robust predictors of negative health outcomes in this population, such as dependence, institutionalization, and death.^(1,2,4,5)^

Functional disability is a common health problem among older people and is generally assessed based on the performance of basic activities of daily living (ADLs) and instrumental activities of daily living (IADLs).^(6)^Although the performance of basic ADLs, which involve self-care and mobility tasks, tends to diminish more slowly in the aging process than the performance of IADLs, individuals in the same age group with different health conditions can have distinct decline trajectories.^(6,7)^

Besides health conditions, sex, age, and poor living conditions have been identified as risk factors for lower levels of strength and physical performance, leading to limitations in performing ADLs, as well as greater levels of dependence and the need for long-term care, with unfavorable consequences for older people, their families, and the community.^(1,5,8)^

Studies have highlighted the importance of identifying functional decline in capacity through grip strength and gait speed assessments. However, the literature has not yet clarified which of these aspects has a stronger association with or greater predictive power for dependence in basic ADLs, especially in populations with distinct demographic and socioeconomic characteristics.^(5,6,8,9)^

In a study involving 242 community-dwelling older people, Bahat et al.^(9)^found that dependence in basic ADLs and IADLs was moderately associated with gait speed (*r*=0.49, *r*=0.63; p<0.001, respectively) grip strength showed a weak correlation with basic ADL and IADL (*r*=0.28, *r*=0.35; p<0.001); however, the skeletal muscle mass index was not correlated with these functionality measures. In a longitudinal study involving 6,217 older people, Zhang et al.^(6)^ found that gait speed had greater discriminatory power than grip strength for the identification of dependence in basic ADLs in men, but not IADLs (area under the receiver operating characteristic (ROC) curve=0.7; 95% confidence interval (CI)=0.66-0.74). Rijk et al.^(5)^ conducted a meta-analysis and found a significant positive association between grip strength decrease and decline in the performance of basic ADLs in five of eight studies analyzed.

However, we found no references in the literature addressing Brazilian older adults in a longitudinal follow-up (excluding participants with total dependence or in at least one ADL at baseline) that tested the associations between functional disability (assessed based on one’s performance on basic ADLs) and biological markers such as strength and gait speed. Therefore, we hypothesized that low muscle strength and gait speed at baseline are predictors of disability in basic ADL.

## OBJECTIVE

To determine whether low muscle strength and gait speed increase the risk of disability related to basic activities of daily living in community-dwelling older adults.

## METHODS

### Study design and local

This longitudinal study was conducted using data from the *Fragilidade em Idosos Brasileiros* (Frailty in Older Brazilians - FIBRA Study). The first data collection was conducted between 2008 and 2009, corresponding to the baseline of the present study. The second collection was conducted between 2016 and 2017, corresponding to this study’s follow-up. Data were obtained from the city of Campinas and the district of Ermelino Matarazzo in the city of São Paulo, both located in southeastern Brazil (detailed information about the sample recruitment strategy, sample characteristics, and main results have been reported previously).^(10)^

### Participants and procedures

The sample included individuals aged ≥65 years residing in urban areas who participated in both the first wave (2008-2009) and follow-up (2016-2017) of the FIBRA Study.^(10)^ In both waves, the data collection procedures lasted an average of 80 minutes and were performed in a single session by previously trained interviewers.

**Figure 1:**
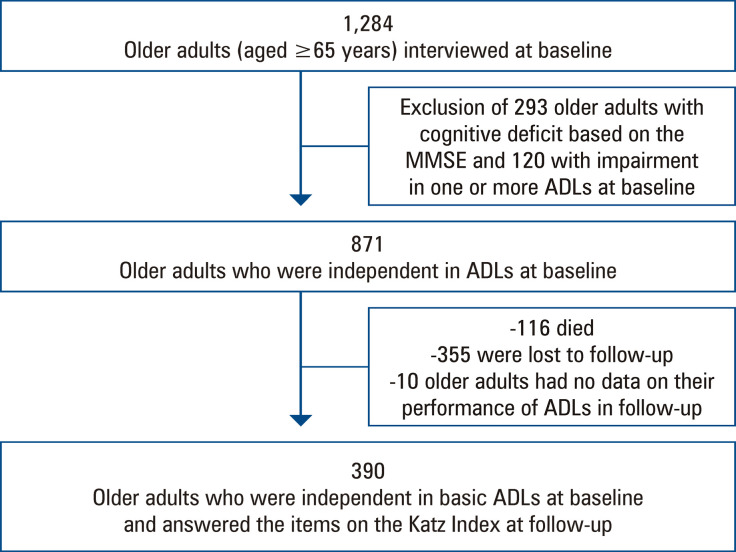
Decisions and procedures for the construction of the sample for this investigation. Data from the *Fragilidade em Idosos Brasileiros* (Frailty in Older Brazilians - FIBRA Study) MMSE: Mini-Mental State Examination; ADLs: activities of daily living.

The design of the baseline study (2008-2009) involved an initial block of sociodemographic, anthropometric, clinical (blood pressure), and frailty measures, as well as screening for dementia, in which all individuals recruited from Campinas and Ermelino Matarazzo (n=1,284) participated.^(10)^ At the end of this phase, those who scored below the cutoff point on the Mini-Mental State Examination^(11)^adjusted for schooling (17 illiterate, 22 with 1-4 years, 24 with 5-8 years, and 26 with ≥9 years of schooling)^(12)^ were excluded (n=293). Individuals who completed the second phase of the baseline study (n=991) participated in an interview addressing health, functioning, and psychosocial aspects. Among these individuals, 120 were excluded because of partial or complete dependence in at least one basic ADL^(13)^ at baseline. Thus, data from 871 individuals were considered eligible for the follow-up study (2016-2017). However, 116 died, and 355 individuals were lost to follow-up (not located or declined to participate). Analysis of the follow-up database revealed that 10 of the 400 individuals interviewed in this phase had no data on their performance of basic ADLs. These individuals were therefore excluded, and the sample of the present study comprised 390 older people who were independent in basic ADLs at baseline and answered the items on the Katz Index at follow-up (Figure 1).^(13)^

### Measures

Functional capacity: The variable of interest in the present study was assessed through self-reports of the participants regarding the performance of basic ADLs on the modified Katz Index (bathing, dressing, toileting, transfers, continence, and feeding).^(13)^ The participants reported whether they needed assistance from others to perform these activities. If they needed assistance, the participants were asked whether they required partial or complete assistance to perform the task. Individuals whose baseline and follow-up records revealed a transition from complete independence to partial or complete dependence on one or more basic ADLs were considered incident cases of disability.

Low muscle strength: Individuals with mean grip strength <27kg for men and <16kg for women, as recommended by the European Working Group on Sarcopenia in Older People (EWGSOP2).^(14)^ Grip strength was measured in both waves of the study using a Jamar dynamometer (Lafayette Instruments, Lafayette, Indiana, United States) on the dominant hand with three trials and a 1-minute rest interval between trials.^(15)^

Slow gait speed: individuals with walking speed ≤0.8m/s^(15)^ indicated by the mean time the participant took to walk at their usual pace a distance of 4 meters, three times.^(16)^

### Covariates

- Sociodemographics: sex (female; male), age group (60-69, 70-79, or ≥80 years), and schooling (never studied; 1-4 years; ≥5 years).

- Lifestyle characteristics: current smoking habits (yes; no), alcohol intake based on the frequency of consumption of alcoholic beverages (never, 1-4 times per month, 2-3 times per week, ≥4 times per week), and physical activity (weekly frequency and daily duration of physical exercise based on answers to the items on the Minnesota Leisure-Time Physical Activity Questionnaire);^(17)^ individuals in the lowest quintile of the sum of metabolic equivalent distribution were classified as inactive in the context of leisure time activities.

Body mass index (BMI), chronic diseases and depressive symptoms: BMI, categorized following the Pan American Health Organization classification: underweight (BMI ≤23kg/m^2^), ideal range (23 <BMI <28kg/m^2^), and overweight/obese (BMI ≥28kg/m^2^);^(18)^ number of chronic noncommunicable diseases (self-report of whether a physician had performed a previous diagnosis [yes; no] of heart disease, hypertension, stroke, diabetes mellitus, cancer, arthritis/rheumatic disease, depression, lung disease, or osteoporosis; subsequently categorized as ≤1 and ≥2 diseases). Depressive symptoms were assessed by using the 15-item Geriatric Depression Scale.^(19)^ The cutoff point for depressive symptoms was > 6.

### Statistical analysis

Descriptive analyses (measures of absolute and relative frequency) were performed to characterize the sample. Percentage distributions were estimated using 95%CI. Associations between low muscle strength, gait speed, and the covariates were determined using Pearson’s χ^2^ test with a 5% significance level. The prevalence of low muscle strength, gait speed, and dependence on ADLs at baseline and follow-up was estimated and the association was verified using Pearson’s χ^2^ test, considering a significance level of 5%. Next, logistic regression analysis was performed to estimate the crude and adjusted odds ratios (OR) and their respective 95%CI. Sex, age group, schooling, and other covariates that presented a p<0.20 in association with the dependent variables were incorporated into the adjusted analysis (for low muscle strength: BMI and depressive symptoms; slow gait speed: physical activity, BMI, alcohol intake, number of diseases, and depressive symptoms). Data analysis was performed using Stata, version 15.0 (Stata Corp., College Station, United States).

### Ethical aspects

The FIBRA study projects were submitted and approved by the Research Ethics Committee of the *Universidade Estadual de Campinas* (CAAE: 49987615.3.0000.5404; 1.332.651) and the *Escola de Artes, Ciências e Humanidades, Universidade de São Paulo* (CAAE: 92684517.5.3001.5390; 2.952.507). All participants and their respective family members received clarifications regarding the objectives and procedures of the study as well as their rights, and informed consent was obtained prior to the interview.

## RESULTS

Among the 390 older adults who were independent at baseline and answered basic ADLs instrument, mean age was 71.7±5.07 years, 66.9% were women, 61.1% were aged ≥70 years, 60.5% had 1-4 years of schooling, 65.0% were physically active, 44.0% were classified as overweight or obese, 90.6% were non-smokers, 4.9% consumed alcohol ≥4 times per week, 67.6% had ≥2 chronic diseases and 17.6% had depressive symptoms. Low muscle strength and lower gait speed were observed in 11.2% and 18.0% of older adults, respectively. No significant differences were observed regarding the comparison of participants and those not included in the follow-up study (p>0.05) (Table 1).

**Table 1:** Characterization of the sample and comparison between the frequencies of older adults interviewed and lost in the follow-up study, considering the independent and adjustment variables for this study. (FIBRA Study, Campinas and Ermelino Matarazzo/SP, 2008-2009 and 2016-2017)

Variables	Participants (n=390) %	Losses (n=354) %	p value*
Sex			
Male	33.1	31.3	0.597
Female	66.9	68.7	
Age group			
65-69 years	39.9	40.9	0.739
70-79 years	53.2	50.4	
≥80 years	7.9	8.7	
Schooling			
None	15.3	14.4	0.401
1-4 years	60.5	57.1	
≥5 years	24.2	28.5	
Physical activity			
Active	65.0	62.5	0.475
Inactive	34.9	37.5	
Body mass index			
Ideal range	40.4	42.8	0.558
Underweight	15.6	13.0	
Overweight/obesity	44.0	44.2	
Smoking habit			
No	90.6	89.8	0.710
Yes	9.4	10.2	
Frequency of alcohol consumption			
Never/to 3 times a week	95.1	94.9	0.886
≥4 times per week	4.9	5.1	
Morbidities			
0-1	32.4	36.2	0.276
≥2	67.6	63.8	
Depressive Symptoms			
No	82.4	83.6	0.670
Yes	17.6	16.4	
Low muscle strength			
No	88.8	86.8	0.393
Yes	11.2	13.2	
Slow gait speed			
No	82.0	80.6	0.603
Yes	18.0	19.4	

* p value of Pearson’s χ^2^ test.

The prevalence of low muscle strength was higher in underweight individuals (19.7%). The prevalence of slow gait speed was higher among women and leisurely inactive individuals (23.9%) (Table 2).

**Table 2:** Prevalence of low muscle strength and low gait speed, according to independent variables at the baseline (FIBRA Study, Campinas and Ermelino Matarazzo/SP, 2008-2009 and 2016-2017)

Variables	Low grip strength		Slow gait speed	
	%	p value	%	p value
Sex		0.851		0.027*
Male	10.8		11.7	
Female	11.5		20.8	
Age group		0.580		0.930
65-69 years	9.8		17.0	
70-79 years	11.6		18.2	
80 years and over	16.1		19.3	
Schooling		0.592		0.137
None	11.7		23.3	
1-4 years	12.3		19.0	
≥5 years	8.4		11.6	
Physical activity		0.836		0.025*
Active	11.1		14.7	
Inactive	11.7		23.9	
Body mass index		0.043*		0.074
Ideal range	7.7		16.1	
Underweight	19.7		9.8	
Overweight/obesity	11.7		22.2	
Smoking habit		0.319		0.241
No	10.8		18.6	
Yes	16.2		10.8	
Frequency of alcohol consumption		0.927		0.143
Never to 3 times a week	11.2		18.5	
≥4 times per week	10.5		5.3	
Morbidities		0.505		0.118
0-1	9.5		13.5	
≥2	11.8		20.0	
Depressive symptoms		0.177		0.200
No	10.3		16.7	
Yes	15.9		23.2	

* Pearson’s χ^2^ p-value: in bold p<0.05 in the association with the independent variables.

**Figure 2:**
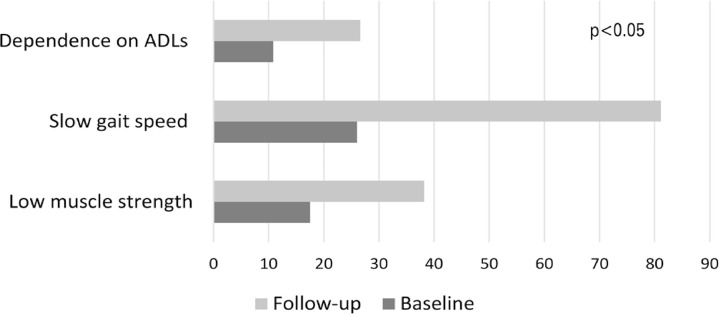
Prevalence of low muscle strength, slow gait speed, and dependence on Basic Activities of Daily Living at baseline and in follow-up. (FIBRA Study, Campinas and Ermelino Matarazzo/SP, 2008-2009 and 2016-2017) ADLs: activities of daily living.

Increases in prevalence between baseline and follow-up were found for low muscle strength (17.5%-38.2%), slow gait speed (26.0%-81.1%), and dependence on basic ADLs (10.8%-26.6%) (Figure 2). The incidence of dependence on basic ADLs was 25.6% (95%CI= 21.5-30.2).

**Figure 3:**
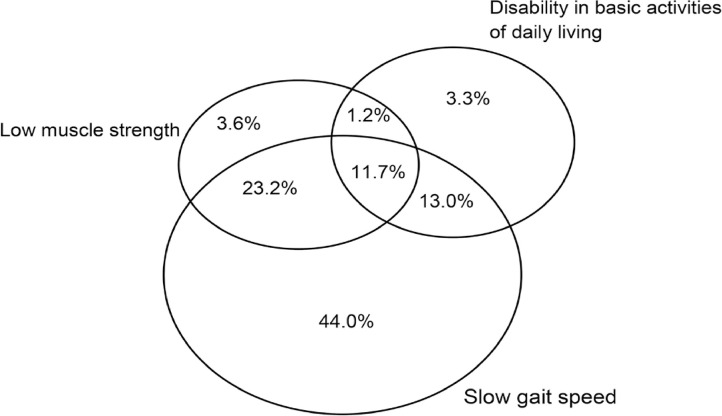
Venn diagram on the isolated and simultaneous presence of slow gait speed (≤0.8m/s), low muscle strength (<27 for men and <16 for women), and disability in one or more basic activities of daily living follow-up (n=332). (FIBRA Study, Campinas and Ermelino Matarazzo/SP, 2008-2009 and 2016-2017)

At follow-up, 11.7% of the participants had concomitant dependence in basic ADLs, slow gait speed, and low muscle strength. Isolated slow gait speed was observed in 44.0% of the participants (Figure 3).

In the unadjusted logistic regression analysis, slow gait speed increased the likelihood of dependence in basic ADLs (OR=2.17; 95%CI=1.25-3.75). In the adjusted model, slow gait speed remained a predictor of dependence on ADLs (OR=1.90; 95%CI=1.06-3.41) (Table 3).

**Table 3:** Logistic regression of dependence in basic activities of daily living according to low muscle strength and low physical performance. (FIBRA Study, Campinas and Ermelino Matarazzo/SP, 2008-2009 and 2016-2017)

Variable	OR unadjusted (95%CI)	OR adjusted (95%CI)
Low muscle strength		
No	1	1
Yes	1.59 (0.81-3.10)	1.65 (0.82-3.29)*
Slow gait speed		
No	1	1
Yes	2.17 (1.25-3.75)	1.90 (1.06-3.41)**

OR: odds ratio; 95%CI: 95% confiance interval.

*Adjusted for low muscle strength: sex; age; schooling; body mass index, and depressive symptoms; **Adjusted for slow gait speed: sex; age; schooling; physical activity; body mass index; alcohol consumption; morbidity and depressive symptoms.

## DISCUSSION

The present longitudinal study aimed to determine whether low muscle strength and lower gait speed increase the risk of disability in performing basic ADLs over a 9-year period. This study stands out for its sample of community-dwelling older people with a high average age at follow-up, conducted in a developing country with considerable diversity in the older population, and the need for a comprehensive long-term care policy focused on functioning in this age group.^(20)^

The data showed an increase in the prevalence of low muscle strength, slow gait speed, and dependence on basic ADLs between baseline and follow-up. Similar results have been previously reported.^(2,8,21)^ A prospective study was conducted with community-dwelling older adults who participated in the Jerusalem Longitudinal Cohort Study (1990-2015) to determine the trajectory of grip strength from the age of 70 to 90 and its association with mood, cognition, functional status, and mortality. Mean grip strength decreased progressively with age, and individuals with low grip strength were at a greater risk of subsequent functional decline and mortality.^(22)^ Another study of older Chinese people found that the decline in gait speed after 4 years was 8.2% for men and 9.0% for women.^(21)^ A prospective cohort composed of 805 community-dwelling Dutch older people (≥60 years) investigated the risk of dependence on basic ADLs, IADLs, and mobility and found that dependence on ADLs, especially household tasks, travelling, shopping, and continence, increased with age.^(23)^

In this study, grip strength was not associated with disability in basic ADLs. However, cross-sectional and longitudinal investigations have found that low muscle strength is associated with a decline in the performance of basic ADLs and IADLs.^(1,24,25)^ A longitudinal study conducted with older adults in Uberaba/MG/Brazil found an association between dependence in basic ADLs and reduced muscle strength (palm grip) after 2 years of follow-up (OR=1.85; 95%CI=1.02-3.33).^(26)^The decline in grip strength, which reflects the diminished function of the neuromuscular system, is associated with poorer performance in ADLs during the aging process.^(1)^

One explanation for this finding is that the participants were healthier than most older people and, therefore, underwent a smaller change in muscle strength (17.5%-38.2%) than in gait speed (26.0%-81.1%) during the period studied. Moreover, greater muscle strength may offer greater protection from the development of dependence in basic ADLs over time.^(2,25,27)^

A reduction in gait speed has been associated with higher rates of disability in basic ADLs and IADLs, as well as sarcopenia, poorer health conditions, hospitalization, and death among older people.^(9)^ Gait speed seems to be a key factor that may mediate the process of disability in basic ADLs. A cross-sectional study using data from the 2015 SABE Study (Health, Well-being, and Aging) involving 19,705 older Columbians with an average age of 70 years found that the association between sarcopenia and functional dependence was mediated by gait speed.^(3)^ In the Uberaba/MG, a longitudinal study of 92 older adults followed up for 4 years after hospital discharge observed an association between changes in IADLs and reduced muscle strength and gait speed.^(28)^ Another study involving 242 older Turks (79.4±5.7 years of age; 31.8% men) recruited prospectively from geriatric outpatient clinics found that functional performance (assessed based on gait speed) was the component of sarcopenia most correlated with basic ADLs and IADLs (p<0.001).^(9)^

In a longitudinal study involving 798 community-dwelling older people who participated in the Invecchiare in Chianti (InCHIANTI) study (n=403) and Dutch participants of the Longitudinal Aging Study Amsterdam (LASA) (n=395), in which the majority reported no functional decline at the onset of the study, Jonkman et al.^(29)^ found three different trajectories of functional decline over a 9-year follow-up: none or little decline, intermediate decline, and severe decline. Although the trajectory courses indicated similar subgroups in men and women, gait speed at baseline was the only consistent predictor of functional decline. A study conducted on a cohort of community-dwelling older French people (n=3,814; 61.0% women) investigated whether gait speed (over 6 m) at baseline and the change in gait speed at follow-up were independently associated with the incidence of functional capacity. The authors found that 16.5% of the participants developed functional disability over the 11-year period, and these individuals had a 20% faster annual decline in gait speed than those without functional decline.^(8)^ These findings are compatible with the data described in the present study, in which low gait speed was the only factor associated with the incidence of dependence in basic ADLs.

Besides being an essential marker of functional capacity in a broad age range,^(2-4,29)^ a reduction in gait speed may also be an early sign of cognitive decline and dementia, as gait is dependent on executive functions.^(27,30)^ Therefore, early identification of the decline in gait performance could prevent not only physical dependence but also the progression of dementia in older people.^(31)^

Measures of muscle strength and gait speed are simple and inexpensive and provide relevant quantitative information in prognostic terms. In the search for practical methods that facilitate the identification of older people at greater risk of disability, gait speed is an easily measurable, clinically interpretable, and potentially modifiable risk factor that has been recognized as a vital sign. Moreover, gait speed is a valid, reliable, and sensitive measure for assessing and monitoring functional status and health in older people.^(15)^

The present study has some limitations, such as a large number of individuals lost to follow-up. Although no statistically significant differences were found between the proportions of individuals who remained and those absent from the follow-up sample, it is possible that the latter group had a greater frequency of chronic diseases and unhealthy behaviors. Another limitation was that intermediate measures were not performed between baseline data collection in 2008-2009 and follow-up in 2016-2017. Another limitation was the use of self-reports for information on functional capacity. However, this instrument is widely used in both research and clinical practice.

## CONCLUSION

In the present study, a greater decline in gait speed was found in the 9-year follow-up period compared to the decrease in muscle strength and occurrence of disability. Moreover, slow gait speed was a predictor of dependence in basic activities of daily living. Thus, gait speed in older people is an important variable for screening functional decline and constitutes an important intervention target. Particularly in primary care, early identification of this outcome could guide the creation of a care plan for older people that involves effective interventions to reverse this condition, aiming to minimize adverse health outcomes, such as dependence, institutionalization, and death. These results can assist healthcare providers in designing directed interventions within a holistic care plan that can contribute to greater levels of autonomy, independence, and well-being in older populations. However, to validate these findings, future studies employing other types of analyses to identify the mediating characteristics of the process of functional disability and the co-occurrence of health conditions that may place older people at greater risk of dependence in basic activities of daily living are needed.
